# Differential Brain Activity in Regions Linked to Visuospatial Processing During Landmark-Based Navigation in Young and Healthy Older Adults

**DOI:** 10.3389/fnhum.2020.552111

**Published:** 2020-10-29

**Authors:** Stephen Ramanoël, Marion Durteste, Marcia Bécu, Christophe Habas, Angelo Arleo

**Affiliations:** ^1^Sorbonne Université, INSERM, CNRS, Institut de la Vision, Paris, France; ^2^Faculty of Psychology and Educational Sciences, University of Geneva, Geneva, Switzerland; ^3^University of Côte d’Azur, LAMHESS, Nice, France; ^4^CHNO des Quinze-Vingts, INSERM-DGOS CIC 1423, Paris, France

**Keywords:** healthy aging, spatial navigation, landmark, fMRI, scene-selective regions

## Abstract

Older adults have difficulties in navigating unfamiliar environments and updating their wayfinding behavior when faced with blocked routes. This decline in navigational capabilities has traditionally been ascribed to memory impairments and dysexecutive function, whereas the impact of visual aging has often been overlooked. The ability to perceive visuospatial information such as salient landmarks is essential to navigating efficiently. To date, the functional and neurobiological factors underpinning landmark processing in aging remain insufficiently characterized. To address this issue, functional magnetic resonance imaging (fMRI) was used to investigate the brain activity associated with landmark-based navigation in young and healthy older participants. The performances of 25 young adults (μ = 25.4 years, *σ* = 2.7; seven females) and 17 older adults (μ = 73.0 years, *σ* = 3.9; 10 females) were assessed in a virtual-navigation task in which they had to orient using salient landmarks. The underlying whole-brain patterns of activity as well as the functional roles of specific cerebral regions involved in landmark processing, namely the parahippocampal place area (PPA), the occipital place area (OPA), and the retrosplenial cortex (RSC), were analyzed. Older adults’ navigational abilities were overall diminished compared to young adults. Also, the two age groups relied on distinct navigational strategies to solve the task. Better performances during landmark-based navigation were associated with increased neural activity in an extended neural network comprising several cortical and cerebellar regions. Direct comparisons between age groups revealed that young participants had greater anterior temporal activity. Also, only young adults showed significant activity in occipital areas corresponding to the cortical projection of the central visual field during landmark-based navigation. The region-of-interest analysis revealed an increased OPA activation in older adult participants during the landmark condition. There were no significant between-group differences in PPA and RSC activations. These preliminary results hint at the possibility that aging diminishes fine-grained information processing in occipital and temporal regions, thus hindering the capacity to use landmarks adequately for navigation. Keeping sight of its exploratory nature, this work helps towards a better comprehension of the neural dynamics subtending landmark-based navigation and it provides new insights on the impact of age-related visuospatial processing differences on navigation capabilities.

## Introduction

The 21st century is characterized by an unprecedented increase in the number of older adults within the worldwide population. There were 703 million people aged 65 years or over in 2019 and this number is projected to more than double by 2050 (United Nations, Departement of Economic and Social Affairs, Population Division, 2019). In parallel, we can expect a significant rise in the prevalence of neurodegenerative diseases such as Alzheimer’s and Parkinson’s diseases in the older population. To identify appropriate biomarkers of age-related sensori-cognitive alterations, it is critical to gain a better understanding of brain changes in healthy aging. In this context, spatial navigation as a complex behavior encompassing perceptual and cognitive processes provides an ideal framework for the study of normal and pathological aging (Gazova et al., 2012; Lithfous et al., 2013; Allison et al., 2016; Laczó et al., 2017, 2018; Coughlan et al., 2018).

An extensive body of literature has highlighted a robust age-related decline in navigation ability in various species including rodents as well as non-human and human primates (Foster et al., 2012; Lester et al., 2017). Healthy older adults exhibit impairments in their capacity to navigate efficiently, reorient or update their wayfinding behavior when faced with obstacles (Iaria et al., 2009; Moffat, 2009; Harris et al., 2012; Daugherty and Raz, 2017; Merhav et al., 2019). In real-world settings, they are impaired at rapidly acquiring information about their surroundings and are thus slower and more error-prone than young adults when navigating (Kirasic, 1991; Wilkniss et al., 1997). In virtual reality (VR) paradigms older adults also choose inefficient routes, underestimate distances, and make frequent turning errors (Adamo et al., 2012). Cross-sectional studies in VR have shed light on an age-related shift in the use of navigation strategies: older adults favor response over place-based strategies (Bohbot et al., 2012; Rodgers et al., 2012). A place-based strategy involves the formation of mental map-like representations of the absolute position of the goal concerning spatial cues in the environment. A response-based strategy refers to the process whereby an association between a specific stimulus and the goal location is formed. The choice of a navigation strategy critically depends on the visual information present in the environment (Foo et al., 2005; Ratliff and Newcombe, 2008). Indeed, successful navigation requires the perception and the integration of relevant visual-spatial cues such as buildings or monuments, and the binding of these salient elements to directional information (Ekstrom, 2015; Epstein et al., 2017; Julian et al., 2018).

Visual-spatial cues can be salient objects used as navigational landmarks or characteristics about the geometric shape of space (Lester et al., 2017; Bécu et al., 2020). Landmarks can be conceptualized as discrete objects that are independent of the environment’s layout, such as a tree or a monument (Epstein and Vass, 2014). Landmarks’ size, stability, and proximity to the goal are among the key factors that influence their use for navigation (Stankiewicz and Kalia, 2007; Auger et al., 2012; Auger and Maguire, 2018). Geometric cues encompass all the elements that are intrinsic to and continuous with the external limits of space. These elements include the overall layout, boundaries of the environment, wall lengths, and angle dimensions (Cheng and Newcombe, 2005; Tommasi et al., 2012; Giocomo, 2016). Several studies in virtual environments have emphasized the idea that old age hinders the ability to use landmark information for navigation (Picucci et al., 2009; Harris et al., 2012; Wiener et al., 2012; Zhong and Moffat, 2016; Hartmeyer et al., 2017). More recently, Bécu et al. (2020) extended these findings by unveiling an age-related preference for geometric cues during real-world navigation, when both types of spatial cues were informative.

Despite the extensive body of literature characterizing the neural underpinnings of human spatial navigation (for recent reviews see Chersi and Burgess, 2015; Spiers and Barry, 2015; Epstein et al., 2017; Herweg and Kahana, 2018; Julian et al., 2018), few experiments have explored this question in the context of healthy aging. To date, only 15 neuroimaging studies have focused on spatial processing in normal aging and the majority of these have used structural analyses (Li and King, 2019). These studies, both cross-sectional and longitudinal, have highlighted an age-related decline in place-based navigation associated with structural changes to the hippocampus mainly (Lövdén et al., 2012; Daugherty et al., 2016; Korthauer et al., 2016; Daugherty and Raz, 2017). Age-related changes in other structures of the medial temporal lobe such as the entorhinal cortex, as well as changes in the prefrontal cortices and cerebellum have also been reported (for recent reviews see Lester et al., 2017; Li and King, 2019). Only one cross-sectional functional magnetic resonance imaging (fMRI) study has investigated the link between the use of visual-spatial cues and the navigational skills of young and older adults (Schuck et al., 2015). The authors combined computational modeling and fMRI during a virtual-navigation task to examine how participants learned object locations relative to a circular enclosure or a salient landmark. Young participants used a hippocampal-dependent system for the representation of geometry (circular arena) and a striatal-dependent system for the representation of a landmark (traffic cone). On the other hand, older participants relied on hippocampal structures for landmark-based navigation and were insensitive to geometric information provided by the environmental boundaries. This absence of reliance on geometric information is surprising considering the behavioral findings mentioned above, and it could be related to the small field of view inside the scanner (Sturz et al., 2013).

Several other brain regions, known to be altered in healthy aging (Lester et al., 2017; Zhong and Moffat, 2018), have also been identified as crucial for visuospatial processing during navigation (Epstein and Vass, 2014; Julian et al., 2018). Recently, there has been growing interest in unearthing the roles of the parahippocampal place area (PPA), the occipital place area (OPA), and the retrosplenial cortex (RSC). These regions are all involved in the processing of visual scenes such as landscapes or urban environments. In particular, they have been speculated to integrate incoming visual inputs with higher-level cognitive processes (for reviews see Epstein et al., 2017; Julian et al., 2018). The PPA is sensitive to navigationally relevant cues (Janzen and van Turennout, 2004; Epstein, 2008) and it may be implicated in the recognition of spatial contexts (Marchette et al., 2015). The OPA has been associated with the processing of local elements in scenes (Kamps et al., 2016) as well as with the representation of environmental boundaries (Julian et al., 2016). The RSC is suggested to play a role in anchoring heading information to local visual cues (see Mitchell et al., 2018 for a recent review of RSC functions). The exploration of the neural activity of these scene-selective regions in the context of aging has just begun. Nevertheless, some evidence exists about functional changes in the PPA and RSC of older adults that have been linked to impaired processing of visual scenes and difficulties in switching between navigation strategies, respectively (Ramanoël et al., 2015; Zhong and Moffat, 2018). The impact of aging on the OPA has not been fully characterized yet, but recent findings have hinted at its preserved connectivity with other navigational brain structures in healthy aging (Ramanoël et al., 2019).

Thus, although several behavioral studies have provided evidence for differential use of landmarks across the lifespan, there is a lack of knowledge on the functional and neurobiological factors responsible for the deterioration of landmark information processing in older age. To address this caveat, the present study used fMRI to investigate to what extent healthy aging influences behavior and neural activity associated with landmark-based navigation. A second objective consisted in deciphering the role played by scene-selective regions (namely, PPA, OPA, RSC) in age-related landmark-based navigation deficits as these areas appear to be critical for the integration of relevant visual information for navigation.

## Materials and Methods

### Participants

Overall, 25 young adults (18 males, seven females) and 21 older adults (eight males, 13 females) completed the experiment, but four older adults were excluded: two (females) for a lack of task understanding and two (one male, one female) for in-scanner motion (movements >5 mm across trials). Thus, 25 young adults (18 males, seven females; 25.4 ± 2.7 years) and 17 older adults (seven males, 10 females; 73.0 ± 3.9 years) were included in the analyses. The participants were part of the French cohort study *SilverSight* (~350 subjects) established in 2015 at the Vision Institute, Quinze-Vingts National Ophthalmology Hospital, Paris (Lagrené et al., 2019). The battery of clinical and functional examinations used to enroll participants comprised an ophthalmological and functional visual screening, a neuropsychological evaluation, an oculomotor screening, an audio-vestibular assessment as well as a static/dynamic balance examination. The neuropsychological evaluation included the Mini-Mental State Examination (MMSE; Folstein et al., 1975) and computerized versions of the 3D mental rotation test (Vandenberg and Kuse, 1978), perspective-taking test (Kozhevnikov and Hegarty, 2001), and forward and backward spans of the Corsi block-tapping task (Corsi, 1973). Enrolled older participants had a score of 24^1^ or higher on the MMSE. All subjects were right-handed, they had a normal or corrected-to-normal vision, and they had no history of neurological or psychiatric disorders. Centration measurements and acuity were evaluated at least 2 weeks before the experimental session to order MRI-compatible glasses for participants requiring visual correction (manufactured by Essilor International). Participants gave their written informed consent to participate in the study. All screening and experimental procedures were compliant with the tenets of the Declaration of Helsinki, and they were approved by the Ethical Committee “CPP Ile de France V” (ID_RCB 2015-A01094-45, CPP N°: 16122).

### Virtual Navigation Task and Experimental Protocols

#### The Virtual Navigation Task

The virtual navigation task was displayed on an MRI-compatible liquid crystal display monitor (NordicNeuroLab, Bergen, Norway) positioned at the head of the scanner bore. Participants viewed the screen [size: 69.84 cm (H) × 39.26 cm (V); pixels: 1,920 × 1,080] at a distance of 115 cm *via* a mirror fixed above the head-coil. The visible part of the screen subtended approximately 34 × 20° of visual angle.

**Figure 1:**
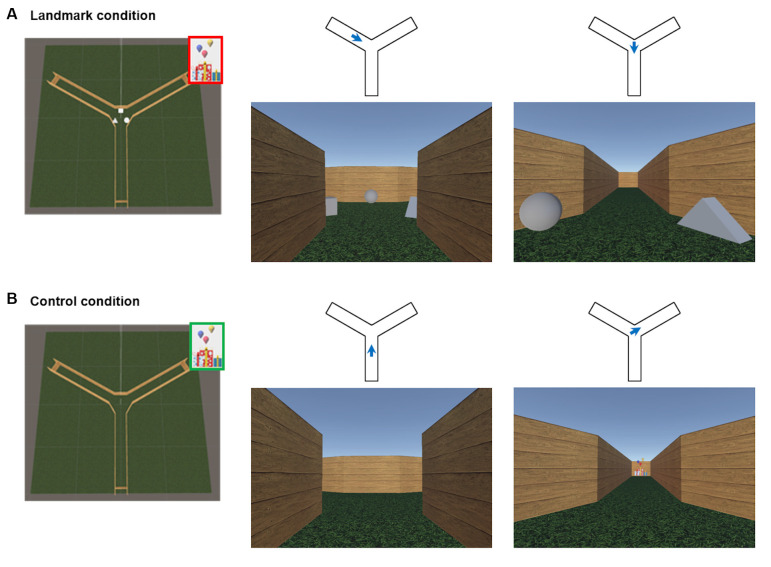
The virtual environment. **(A)** An overhead perspective of the environment for the landmark condition and two example views representing a first-person perspective within the maze. Blue arrows represent the position and the orientation associated with the example views in the landmark condition. **(B)** An overhead perspective of the environment for the control condition and example views within the maze. Blue arrows represent the position and the orientation associated with the example views in the control condition. Red: hidden goal; Green: visible goal. The aerial view was never seen by participants.

The virtual environment was programmed with the Unity3D game engine (Unity Technologies SF; San Francisco, CA, USA^2^) and it allowed participants to navigate actively from a first-person perspective. The virtual environment was a three-arm maze (Y-maze) consisting of three corridors radiating out from a center delimited by homogenous wooden-like walls. Two configurations were designed. In the *landmark condition*, all arms were 18 virtual meters (vm) long and equiangular. Three 3D light gray-colored objects (a square, a triangle, and a circle) were placed in between the arms at the center of the maze (Figure 1A). In the *control condition*, the arms were still 18 vm long and equiangular, but the maze was devoid of objects (Figure 1B).

Participants navigated actively through the virtual environment with an MRI-compatible ergonomic two-grip response device (NordicNeuroLab, Bergen, Norway). They could move forward (thumb press), turn right (right index press), and turn left (left index press). A single finger press was necessary to initiate or stop the movement. The forward speed of movement was set at 3 vm/s and the turning speed at 40°/s.

#### The Spatial Navigation Paradigm

Before scanning, all participants familiarized themselves with the response device in an unrelated virtual space both outside and inside the scanner. They were required to navigate within a square open-field environment and to walk over a wooden board that appeared at different locations.

The scanning session during the navigation task was divided into three runs: an encoding phase and a retrieval phase for the landmark condition and a control condition. At the beginning of the *encoding phase*, participants were positioned at the center of the maze randomly facing one of the three arms. They were instructed to find a goal (gifts) hidden at the end of one corridor and to remember its location using the visual information available in the center of the environment (i.e., the three 3D light gray-colored objects; Figure 1A). The goal appeared when the subject arrived at the correct location. The encoding phase lasted 3 min to ensure that participants could explore all corridors. Then, the *retrieval phase* began and it consisted of seven trials. In each trial, participants were placed at the end of a non-rewarded corridor with their back against the wall. The starting positions were pseudo-randomized across both trials and subjects. Participants were asked to navigate to the previously encoded goal location. Upon arrival at the end of the correct arm, the gifts appeared to indicate successful completion of the trial, and a fixation cross on a gray screen was presented for an inter-trial interval of 3–8 s. Finally, the control condition began and it included four trials. At the beginning of each control trial, subjects started at the end of an arm and moved to the center of the maze. The target was readily visible from the center and participants were instructed to navigate towards it. The control condition was designed to account for potential confounding factors such as motor and simple perceptual aspects of the task.

A short debriefing phase concluded the experimental session. Participants were probed on the strategy they used to orient in the landmark condition. They were asked to report how they solved the task: (i) using one object; (ii) using at least two objects; (iii) randomly; and (iv) another strategy. Participants were deemed to be using a *place-based* strategy when their decision was based on two landmarks or more and to be using a *response-based* strategy when their decision was based on a single visual-spatial cue (Iaria et al., 2003; Iglói et al., 2010, 2015; Chrastil, 2013; Gazova et al., 2013; Packard and Goodman, 2013; Colombo et al., 2017; Laczó et al., 2017). No participants answered that they oriented randomly or that they used a different strategy.

#### Functional Localizer Experiment

Following the spatial navigation task, a block fMRI paradigm similar to that used by Ramanoël et al. (2019) was used to locate the scene-selective regions: the PPA, the OPA, and the RSC. Participants were presented with blocks of 900 × 900-pixel grayscale photographs (18 × 18° of visual angle) representing scenes, faces, everyday objects, and scrambled objects. The functional run lasted 4 min 40 s and it was composed of 14 20-s task blocks (four blocks of scenes, two blocks of faces, two blocks of objects, two blocks of scrambled objects, and four blocks of fixation). Each stimulus was presented for 400 ms followed by a 600 ms inter-stimulus interval. Participants performed a “one-back” repetition detection task.

#### Statistical Analyses of Behavioral Data

During all phases of the virtual-navigation task, the trial durations (to calculate the average navigation time, i.e., the mean time to reach the goal), the grip responses (i.e., the number of times each button of the MRI-compatible two-grip response device was pressed), and the error rate (i.e., the number of times a participant chose the wrong corridor across the seven trials) were recorded. These behavioral measures were used to quantify the spatial navigation performance of subjects, which was compared across age group and sex. The normality of the behavioral data was assessed graphically with quantile-quantile plots and numerically with the Shapiro–Wilk test. Descriptive characteristics and behavioral measures were compared using independent samples *t*-test for normally distributed continuous data, Mann–Whitney *U* tests for non-normally distributed continuous data, and chi-square test of independence for categorical data. To investigate the potential influence of strategy use on navigation performance, simple logistic regressions were conducted in both age groups. Also, the links between sex and strategy use were examined with Fisher’s exact test. Finally, associations between neuropsychological test scores and behavioral performance were computed using simple linear regression analyses with a statistical threshold set at *p* < 0.005 after adjusting for multiple comparisons [*p* = 0.05/(5 × 2)].

### Acquisition and Statistical Analyses of MRI Data

#### MRI Acquisition

Data were collected using a 3 Tesla Siemens MAGNETOM Skyra whole-body MRI system (Siemens Medical Solutions, Erlangen, Germany) equipped with a 64-channel head coil at the Quinze-Vingts National Ophthalmology Hospital in Paris, France. T2*-weighted echo-planar imaging (EPI) sequences, optimized to minimize signal dropout in the medial temporal region (Weiskopf et al., 2006), were acquired for functional imaging during the navigation task (voxel size = 3 × 3 × 2 mm, TR/TE/flip angle = 2685 ms/30 ms/90°, interslice gap = 1 mm, slices = 48, matrix size = 74 × 74, FOV = 220 × 220 mm). For the localizer experiment, 284 volumes from 64 slices were acquired using a T2*-weighted simultaneous multi-slice echo planar sequence (SMS-EPI; voxel size = 2.5 × 2.5 × 2.4 mm, TR/TE/flip angle = 1,000 ms/30 ms/90°, matrix size = 100 × 100, SMS = 2, GRAPPA = 2). The anatomical volume consisted of a T1-weighted, high-resolution three-dimensional MPRAGE sequence (voxel size = 1 × 1 × 1.2 mm, TR/TE/IT/flip angle = 2,300 ms/2.9 ms/900 ms/9°, matrix size = 256 × 240 × 176).

#### Whole-Brain Analyses

FMRI data analysis was performed using a combination of SPM12 release 7487 (Wellcome Department of Imaging Neuroscience, London, UK) and ArtRepair toolbox (Mazaika et al., 2009) implemented in MATLAB 2015 (Mathworks Inc., Natick, MA, USA). The first five functional volumes of the encoding, retrieval, and control runs were discarded to allow for equilibration effects. A slice-timing correction was applied and functional images were realigned to the mean functional image using a rigid body transformation. Artifacts related to the motion were then examined with ArtRepair. Volumes displaying elevated global intensity fluctuation (>1.3%) and movement exceeding 0.5 mm/TR were repaired using interpolation from adjacent scans. The T1-weighted anatomical volume was then realigned to match the mean functional image of each participant and normalized to the Montreal Neurological Institute (MNI) space using a 4th-degree B-Spline interpolation. The anatomical normalization parameters were subsequently used for the normalization of functional volumes. Each functional scan was smoothed with an 8 mm FWHM (Full Width at Half Maximum) Gaussian kernel. The preprocessed images were visually inspected to ensure that there were no realignment or normalization issues.

Statistical analysis was performed using the general linear model for block design at the single-participant level (Friston et al., 1995). The seven trials of the retrieval phase in the landmark condition, the four trials of the control condition, and fixation times were modeled as regressors, constructed as box-car functions, and convolved with the SPM hemodynamic response function (HRF). The encoding phase was not included in the analysis as the time taken to find the goal for the first time differed greatly both within and between age groups. Time to reach the goal by trial, grip responses during navigation, and movement parameters derived from the realignment correction (three translations and three rotations) were entered in the design matrix as regressors of no-interest. The time series for each voxel was high-pass-filtered (1/128 Hz cutoff) to remove low-frequency noise and signal drift. Individual contrasts were submitted to multiple regression and a two-samples *t*-test. The main contrast of interest for all analyses was [Landmark > Control]. Sex and total brain volume (gray and white matter) were included as covariates in the regression and the total brain volume was included as a covariate in the two-samples *t*-test (see “Behavioral Results” section). Areas of activation were tested for significance using a statistical threshold of *p* < 0.001 uncorrected at voxel-level, with a minimum cluster extent of *k* = 10 voxels (Iglói et al., 2010, 2015; Sutton et al., 2010; Schuck et al., 2015; Javadi et al., 2017).

Of note, a control analysis that excluded all error trials was performed to identify the potential impact of errors on variability in brain activity. No significant change to the fMRI results was observed after removing error trials.

### Region-of-Interest Analyses

Data from the localizer experiment were analyzed using SPM12. For each participant, the first four functional localizer volumes were discarded and the remaining images were realigned, co-registered to the T1-weighted anatomical image, normalized to the MNI space, and smoothed using an 8 mm FWHM Gaussian kernel. The slice-timing correction was not applied, following recommendations from the Human Connectome Project functional preprocessing pipeline for multi-slice sequences (Glasser et al., 2013). The localizer images were analyzed using a single participant general linear model for block design. Five categories of interest (scenes, faces, objects, scrambled objects, fixation) were modeled as five regressors and convolved with a canonical HRF. Movement parameters were included in the model as regressors of no interest and each voxel’s time-series was high-pass-filtered (1/128 Hz cutoff).

PPA, OPA, and RSC were located independently for each participant using the fMRI contrast [Scenes > (Faces + Objects)]. Significant voxel clusters on individual *t*-maps were identified using family-wise error correction (FWE) for multiple comparisons (α = 0.05, *t*-value > 4.8). Mask ROIs were created as the 40 contiguous voxels with the highest *t-values* around the peaks of activation from the left and right hemispheres. The two 40-voxel regions from each hemisphere were subsequently summed into a single 80-voxel ROI. Mean parameter estimates were extracted from the three mapped ROIs using the REX MATLAB-based toolkit. Analogously to the whole-brain analyses, the main contrast of interest for the ROI analyses was [Landmark > Control].

## Results

### Behavioral Results

**Figure 2:**
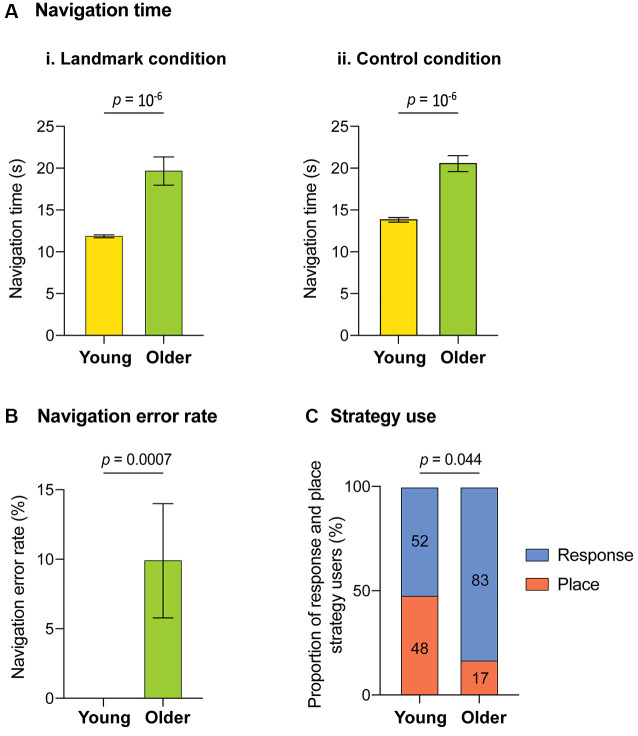
Behavioral results for the virtual-navigation task across age groups. **(A)** Time taken to reach the goal (navigation time): **(i)** averaged across seven trials in the landmark condition, **(ii)** averaged across four trials in the control condition. **(B)** Time taken to reach the goal averaged across seven trials in the landmark condition (navigation time). **(C)** The proportion of participants who used a place-based or response-based strategy in the landmark condition (strategy use). Error bars represent the standard errors of the mean.

The navigation performances across age groups are presented in Figure 2. Older subjects were significantly slower to reach the goal than younger subjects (i.e., longer navigation time) in the landmark condition (19.85 s ± 1.67 vs. 11.97 s ± 0.13; *U*_(40)_ = 418, *p* = 10^−6^, *r* = 0.81, 95% confidence interval (CI) of the difference [3.40, 8.36]; Figure 2Ai) and in the control condition (20.83 s ± 0.95 vs. 14.07 s ± 0.27; *U*_(40)_ = 418, *p* = 10^−6^, *r* = 0.81, 95% CI of the difference [4.70, 8.15]; Figure 2Aii). In addition, older adults made more navigation errors than young adults (mean ± SEM: 1.0 ± 0.41 vs. 0 ± 0.00; *U*_(40)_ = 125, *p* = 0.0007, *r* = 0.44, 95% CI of the difference [0.00, 0.00]). On average, older adults chose the wrong corridor in 10% of trials (Figure 2B). No sex effect was observed on navigation time in each age group separately. However, when data from both age groups were pooled, women’s navigation time was significantly longer than men’s (17.75 s ± 1.88 s vs. 13.40 s ± 0.64 s; *U*_(40)_ = 2.29, *p* = 0.022, *r* = 0.35, 95% CI of the difference [0, 15, 7.05]). Sex and total intracranial volume were therefore included as covariates in the fMRI multiple regression analyses. The post-scanner debriefing phase revealed a significant difference in the type of navigational strategy used between age groups ($X_{\left(1,N=42\right)}^{2}$ = 4.06, *p* = 0.044, *φ* = 0.67). Indeed, older adults relied less on place strategies during landmark-based navigation than younger adults (Figure 2C). However, in neither age group did strategy use predict navigation performance. Finally, there was no sex effect on strategy use.

Neuropsychological assessments showed that older adults had significantly poorer performance than young adults across all measures. Descriptive and cognitive characteristics are summarized in Table 1. Finally, longer navigation time was associated with poorer performance on the forward span of the Corsi block-tapping task (*R*^2^ = 0.31, *p* = 0.0001, *f*^2^ = 0.45), on the backward span of the Corsi block-tapping task (*R*^2^ = 0.19, *p* = 0.004, *f*^2^ = 0.23) and on the perspective-taking test (*R*^2^ = 0.55, *p* = 10^−8^, *f*^2^ = 1.22). A significant association was also reported between the number of errors during the virtual-navigation task and performance on the perspective-taking test (*R*^2^ = 0.58, *p* = 10^−9^, *f*^2^ = 1.38).

**Table 1:** Descriptive characteristics and cognitive performance of young and older participants.

	Groups				
	Mean (± SEM)				
Sex (M/F)	Young 18/7	Older 7/10	*p*-value	ES*	95% CI of the difference
Age^1^	25.4 (± 0.5)	73.0 (± 0.9)	*p* < 0.001	14.8	[45.6, 49.7]
Total brain volume^1^ (cm^3^)	1301 (± 18)	1061 (± 23)	*p* < 0.001	−2.67	[−297.6, −183.3]
MMSE^2^	30.0 (± 0.0)	28.8 (± 0.4)	*p* < 0.001	−0.61	[−2.0, −0.0]
3D mental rotation^1^	18.3 (± 0.9)	12.7 (± 1.2)	*p* < 0.001	−1.20	[−8.8, −2.7]
Corsi forward^2^	7.2 (± 0.2)	4.4 (± 0.2)	*p* < 0.001	−0.80	[−4.0, −2.0]
Corsi backward^2^	6.2 (± 0.3)	4.6 (± 0.2)	*p* < 0.001	−0.54	[−2.0, −1.0]
Perspective taking^2^	15.3 (± 1.7)	46.1 (± 6.7)	*p* < 0.001	0.65	[16.8, 35.7]

M, male; F, female; SEM, standard error of the mean; ES, effect size (*Hedges’ g was computed for independent samples *t*-tests and r was computed for Mann–Whitney U tests); CI, confidence interval (95% CI of the difference between means was computed for independent samples *t*-tests and 95% CI of the difference between medians for Mann–Whitney U tests); MMSE, mini-mental state examination; ^1^independent samples *t*-test; ^2^Mann–Whitney U test.

### Whole-Brain fMRI Results

#### Multiple Regression Analyses

**Figure 3:**
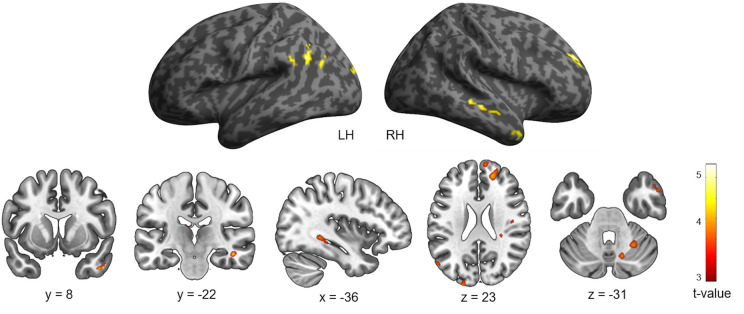
Cerebral regions whose activity for the contrast [Landmark > Control] was predicted by navigation time projected onto 3D inflated anatomical templates and 2D slices (*p* < 0.001 uncorrected, *k* = 10 voxels). LH, left hemisphere; RH, right hemisphere.

The brain regions related to navigation performance were located by examining the association between both groups’ navigation time, age, and patterns of brain activity for the fMRI contrast [Landmark > Control]. A negative association was observed between navigation time and neural activity in multiple clusters across the brain (Table 2 and Figure 3) including frontal (right superior and middle gyri), temporal (middle, inferior, lingual, and parahippocampal gyri), parietal (angular gyrus including the inferior parietal lobule), and occipital cortices (left superior occipital gyrus) as well as the cerebellum (lobule VI and vermis). Temporal activations in the left hemisphere (LH) comprised the posterior part of the hippocampus (*x* = −24, *y* = −46, *z* = 5). Other activations included the ventral temporal cortex (*x* = 45, *y* = 8, *z* = −37) and the visual area V3A (*x* = −21, *y* = −97, *z* = 23), which is part of the dorsal visual stream. The inverse association did not elicit any significant brain activations. Complementary analyses revealed a negative association between age and patterns of neural activity in the left superior temporal gyrus (*x* = −45, *y* = −1, *z* = −16) and the brainstem (*x* = 3, *y* = −7, *z* = −1) for the fMRI contrast [Landmark > Control; Table 2].

**Table 2:** Cerebral regions whose activity for the contrast [Landmark > Control] was predicted by navigation time across all participants and across age groups (sex and intracranial volume were included as covariates).

	H	BA	*k*	*x*	*y*	*z*	*t*	*R*^2^	ES [95% CI]
**Multiple Regression Navigation time/[Landmark > Control]**
Inferior temporal gyrus	R	21	40	45	−22	−16	5.08	0.33	0.24 [0.16, 0.32]
				51	−31	−13	4.80		0.28 [0.19, 0.38]
Lingual gyrus	L	-	27	−30	−58	5	4.69	0.14	0.40 [0.26, 0.54]
				−24	−46	5	4.66		0.46 [0.30, 0.62]
Lingual gyrus	R	-	15	30	−49	2	4.64	0.19	0.54 [0.35, 0.73]
[Middle temporal gyrus]				42	−49	5	3.65		0.22 [0.12, 0.32]
Middle frontal gyrus	R	9	35	18	44	26	4.59	0.09	0.19 [0.13, 0.26]
Cerebellum Vermis	R	-	67	3	−55	−4	4.59	0.15	0.39 [0.25, 0.53]
				3	−43	−13	3.92		0.40 [0.24, 0.57]
Middle temporal gyrus	R	20	10	45	8	−37	4.56	0.29	0.23 [0.15, 0.32]
Parahippocampal gyrus	L	19	13	−33	−40	−4	4.47	0.16	0.36 [0.23, 0.49]
Middle frontal gyrus	R	10	23	15	65	20	4.37	0.09	0.65 [0.41, 0.90]
[Superior frontal gyrus]				6	68	14	3.75		0.61 [0.34, 0.88]
Cerebellum lobule VI	R	-	13	27	−55	−31	4.26	0.24	0.20 [0.13, 0.28]
Superior occipital gyrus	L	18	11	−21	−97	23	4.26	0.11	0.50 [0.31, 0.70]
Angular gyrus	L	39	53	−54	−61	29	4.01	0.22	0.53 [0.31, 0.75]
				−45	−79	35	4.00		0.37 [0.22, 0.52]
				−54	−70	26	3.94		0.30 [0.18, 0.43]
Angular gyrus	R	39	13	45	−58	32	4.01	0.11	0.51 [0.30, 0.72]
Superior frontal gyrus	L	8	10	−9	35	47	3.97	0.13	0.22 [0.13, 0.31]
**Multiple Regression Age/[Landmark > Control]**
Superior temporal gyrus	L	22	31	−45	−1	−16	5.61	0.26	0.16 [0.12, 0.21]
		38		−51	8	−16	3.54		0.08 [0.05, 0.12]
Brainstem	R	-	17	3	−7	−1	5.40	0.11	0.20 [0.14, 0.26]

*The statistical threshold was defined as *p* < 0.001 uncorrected for multiple comparisons at voxel-level with an extent voxel threshold set at 10 voxels. For each cluster, the region with the maximum t-value is listed first and other regions in the cluster are listed [in square brackets]. Montreal Neurological Institute (MNI) coordinates (x, y, z) of the peak activation and the number of voxels (k) in a cluster are also shown. All values including t-values, effect sizes, and 95% confidence intervals were provided for the peak voxel coordinate but the *R*^2^ values were provided for the entire cluster. H, hemisphere; R, right hemisphere; L, left hemisphere; BA, Brodmann area; ES, effect size; CI, confidence interval*.

#### Two-Sample Analyses

**Figure 4:**
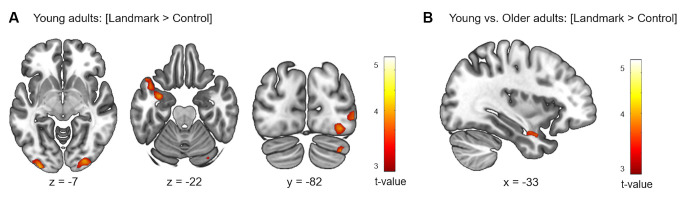
Cerebral regions whose activity for the contrast [Landmark > Control] was elicited by within- **(A)** or between-group **(B)** analyses projected onto 2D slices (*p* < 0.001 uncorrected, *k* = 10 voxels).

First, the brain activity elicited by the contrast [Landmark > Control] was examined in each age group individually. In a second step, between-group analyses were conducted to compare activity between young and older participants. Results for the within-group and between-group analyses are shown in Table 3 and Figure 4. For young participants, significant clusters were found in the superior and inferior temporal gyri of the LH which included the amygdala and hippocampus, in the middle and inferior occipital gyri bilaterally, and in the right cerebellum (Crus I-II). In the older participant group, no significant activation was elicited by the fMRI contrast [Landmark > Control]. Through the direct comparisons between age groups, significant results were found for the fMRI contrast [Young > Older] but not for the contrast [Older > Young]. Young participants exhibited stronger activity in the left inferior temporal gyrus, comprising the amygdala and hippocampal regions, compared to older subjects.

**Table 3:** Cerebral regions whose activity for the contrast [Landmark > Control] was elicited by within-group or between-group analyses (total intracranial volume was included as a covariate).

Group analyses [Landmark > Control]		H	BA	*k*	*x*	*y*	*z*	*t*	ES [95% CI]
**Within-group**
**[Young]**	Inferior Occipital Gyrus	R	18	65	30	−88	−10	5.07	4.49 [3.04, 5.95]
	Superior Temporal Gyrus	L	38	21	−48	20	−25	4.54	2.55 [1.63, 3.47]
					−42	11	−22	4.01	2.52 [1.49, 3.55]
	Cerebellum Crus I-II	R	-	19	33	−82	−34	4.49	2.75 [1.74, 3.76]
	Middle Occipital Gyrus	L	18	37	−30	−97	−7	4.42	3.38 [2.12, 4.64]
	[Inferior Occipital Gyrus]		19		−39	−85	−13	3.54	3.25 [1.74, 4.76]
	Inferior Temporal Gyrus	L	53/54	15	−30	−1	−22	4.37	1.43 [0.89, 1.97]
	(Amygdala/Hippocampus)
	Middle Occipital Gyrus	R	19	16	48	−79	2	4.20	1.85 [1.13, 2.57]
[**Older**]	*No significant activation*
**Between-group**
[**Young > Older**]	Inferior Temporal Gyrus	L	-	12	−33	2	−25	3.86	2.49 [1.43, 3.56]
	(Amygdala/Hippocampus)				−36	−7	−25	3.82	2.13 [1.21, 3.05]
[**Older > Young**]	*No significant activation*								

*The statistical threshold was defined as *p* < 0.001 uncorrected for multiple comparisons at voxel-level with an extent voxel threshold set at 10 voxels. For each cluster, the region with the maximum t-value is listed first and other regions in the cluster are listed [in square brackets]. Montreal Neurological Institute (MNI) coordinates (x, y, z) of the peak activation and the number of voxels (k) in a cluster are also shown. H, hemisphere; R, right hemisphere; L, left hemisphere; BA, Brodmann area; ES, effect size; CI, confidence interval*.

The absence of activation in the older adult group prompted us to conduct further exploratory analyses. First, the cognitive cost of the control condition was investigated both between and within-age groups using the fMRI contrast [Control > Fixation] with a conservative cluster-level FWE correction *p* < 0.05. The latter statistical threshold is applied here because the navigational condition is not compared to an active control condition (i.e., that involves virtual spatial navigation). No significant activation was found for the group comparison [Young > Older]. However, by using the inverse group comparison [Older > Young] activations were revealed in the left superior and middle frontal gyri (Table 4). Within-group analyses revealed a widespread pattern of activity in young adults encompassing the superior occipital, superior frontal, and superior parietal gyri. In older adults, similar activations were noted along with some lingual and precentral activity (Supplementary Table 1). Of note, we observed smaller clusters of activation in the older adult group compared with the younger adult group. Secondly, between- and within-group analyses using the fMRI contrast [Landmark > Fixation] were performed with the same cluster-level FWE correction *p* < 0.05. Significant activations were reported for the [Older > Young] comparison only, and they included the middle frontal and angular gyri in each hemisphere as well as the right cerebellum (Table 5). Concerning the within-group analyses, young participants displayed activity in the middle frontal, superior parietal, and occipital gyri while older participants showed activations of the superior and middle frontal gyri, superior parietal gyrus, precentral gyrus, fusiform gyrus, and cerebellum (Supplementary Table 2).

**Table 4:** Cerebral regions whose activity for the contrast [Control > Fixation] was elicited by between-group analyses (total intracranial volume was included as a covariate).

Group analyses [Control > Fixation]		H	BA	*k*	*x*	*y*	*z*	*t*	ES [95% CI]
**[Young > Older]**	*No significant activation*								
**[Older > Young]**	Superior Frontal Gyrus	L	-	30	−27	32	56	5.56	3.18 [2.06, 4.30]
	[Middle Frontal Gyrus]		8		−36	23	56	4.58	2.44 [1.39, 3.48]

*The statistical threshold was defined as *p* < 0.05 FWE-corrected for multiple comparisons at cluster-level with an extent voxel threshold set at 10 voxels. For each cluster, the region with the maximum t-value is listed first and other regions in the cluster are listed [in square brackets]. Montreal Neurological Institute (MNI) coordinates (x, y, z) of the peak and number of voxels (k) of clusters are also shown. H, hemisphere; R, right hemisphere; L, left hemisphere; BA, Brodmann area; ES, effect size; CI, confidence interval*.

**Table 5:** Cerebral regions whose activity for the contrast [Landmark > Fixation] was elicited by between-group analyses (total intracranial volume was included as a covariate).

Group analyses [Landmark > Fixation]		H	BA	*k*	*x*	*y*	*z*	*t*	ES [95% CI]
**[Young > Older]**	*No significant activation*								
**[Older > Young]**	Middle Frontal Gyrus	R	10	22	24	47	−1	6.06	0.95 [0.69, 1.21]
	Angular Gyrus	L	7	223	−30	−64	47	5.66	2.43 [1.72, 3.14]
	[Superior Parietal Gyrus]		39		−33	−55	44	5.59	2.26 [1.59, 2.93]
	[Supramarginal Gyrus]		40		−48	−43	44	5.32	1.61 [1.11, 2.11]
	Middle Frontal Gyrus	R	10	34	39	38	17	5.37	1.51 [1.05, 1.97]
					33	47	20	4.47	1.40 [0.88, 1.92]
	Cerebellum	R	-	23	36	−73	−22	5.19	2.90 [1.98, 3.82]
									
	Middle Frontal Gyrus	L	6	127	−42	5	41	5.14	1.78 [1.21, 2.35]
			9		−45	26	26	5.13	1.60 [1.09, 2.11]
			6		−45	5	50	5.12	1.73 [1.18, 2.29]
	Superior Parietal Gyrus	R	39	36	30	−55	44	4.89	1.87 [1.24, 2.50]
	[Angular Gyrus]		7		30	−64	53	4.64	3.34 [2.16, 4.53]

*The statistical threshold for the cluster was defined as *p* < 0.05 FWE-corrected for multiple comparisons at the cluster-level with an extent voxel threshold set at 10 voxels. For each cluster, the region with the maximum t-value is listed first and other regions in the cluster are listed [in square brackets]. Montreal Neurological Institute (MNI) coordinates (x, y, z) of the peak and number of voxels (k) of clusters are also shown. H, hemisphere; R, right hemisphere; L, left hemisphere; BA, Brodmann area; FEW, family-wise error; ES, effect size; CI, confidence interval*.

#### Association Between Neuropsychological Evaluation and fMRI Activity

Exploratory regression analyses were also conducted to test the associations between cognitive scores and brain activations for the contrast [Landmark > Control] across all participants. Significant associations were found for the 3D mental rotation test, the forward span of the Corsi block-tapping task, and the perspective-taking test. Specifically, higher scores on the 3D mental rotation test were correlated with enhanced activity in the brainstem (*x* = 9, *y* = −25, *z* = −22; effect size = 0.20, 95% CI [0.13, 0.27]; *R*^2^ = 0.05). Better visuo-spatial working memory was associated with increased activity in the left parahippocampal gyrus and the posterior part of the hippocampus (*x* = −24, *y* = −31, *z* = 16; 1.06 [0.77, 1.36]; *R*^2^ = 0.17). Finally, perspective-taking ability was associated with activation of the superior frontal gyrus bilaterally (left: *x* = −9, *y* = −10, *z* = 83; 0.21 [0.14, 0.28]; *R*^2^ = 0.13; right: *x* = 15, *y* = −13, *z* = 83; 0.26 [0.17, 0.35]; *R*^2^ = 0.13), the left caudate nucleus (*x* = −18, *y* = 14, *z* = 23; 0.07 [0.05, 0.10]; *R*^2^ = 0.29), and the brainstem (*x* = 3, *y* = −25, *z* = −46, 0.21 [0.13, 0.29]; *R*^2^ = 0.21). Of note, effect sizes (ESs) and 95% CIs were computed for the peak voxel coordinate but the *R*^2^ values were computed for the entire cluster.

### Region-of-Interest Results

**Figure 5:**
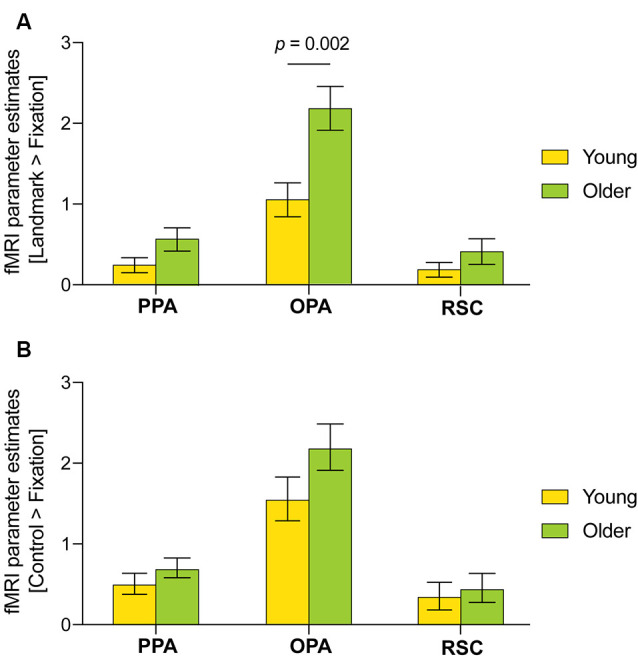
Functional magnetic resonance imaging (fMRI) parameter estimates in the parahippocampal place area (PPA), occipital place area (OPA), and retrosplenial cortex (RSC) for the fMRI contrasts **(A)** [Landmark > Fixation] and **(B)** [Control > Fixation] across age groups. A three-way ANOVA was performed with scene-selective region, age and condition as factors. Only significant differences between age groups are displayed. Error bars reflect standard errors of the mean.

The PPA, OPA and RSC ROIs were defined for each individual based on the independent localizer experiment. First, age-related effects were examined in average parameter activity for the fMRI contrast [Landmark > Control]. No significant differences were found. It is nonetheless interesting to note the presence of OPA activity in older adults only (1.40 ± 1.20 vs. −0.18 ± 0.44, *p* = 0.234; Supplementary Figure 1). Considering the latter result and the age-related differences observed during the control condition, further exploratory analyses were performed using the fMRI contrasts [Landmark > Fixation] and [Control > Fixation]. A three-way ANOVA was conducted with scene-selective region, age and condition as factors using the fMRI contrasts [Landmark > Fixation] and [Control > Fixation] (Figures 5A,B). Scene-selective region (*F*_(2,240)_ = 64.62, *p* < 0.001, partial η^2^ = 0.35, 95% CI [0.26, 0.43]) and age (*F*_(1,240)_ = 16.13, *p* < 0.001, partial η^2^ = 0.06 [0.02, 0.13]) but not condition had an effect on fMRI parameter activity. Moreover, a significant interaction between scene-selective region and age was uncovered (*F*_(2,240)_ = 4.18, *p* = 0.016, partial η^2^ = 0.03 [0.00, 0.09]). *Post hoc* tests revealed that the OPA was more activated than the PPA during the landmark condition in young (1.07 ± 0.21 vs. 0.25 ± 0.09, *p* = 0.040, Hedges’ *g* = −0.91, 95% CI [−1.50, −0.33]) and older adults (2.19 ± 0.27 vs. 0.60 ± 0.14, *p* < 0.001, Hedges’ *g* = −1.52 [−2.28, −0.76]) and during the control condition in young (1.55 ± 0.27 vs. 0.51 ± 0.13, *p* = 0.001, Hedges g’ = −0.81 [−1.38, −0.23]) and older adults (2.19 ± 0.29 vs. 0.70 ± 0.12, *p* < 0.001, Hedges’ *g* = −1.15 [−1.19, −0.43]). The OPA was also significantly more activated than the RSC during the landmark condition in young (1.07 ± 0.21 vs. 0.21 ± 0.09, *p* = 0.022, Hedges’ *g* = −0.83 [−1.41, −0.25]) and older adults (2.19 ± 0.37 vs. 0.43 ± 0.15, *p* < 0.001, Hedges’ *g* = −1.72 [−2.50, −0.93]) and during the control condition in young (1.55 ± 0.27 vs. 0.34 ± 0.17, *p* < 0.001, Hedges’ *g* = −0.99 [−1.58, −0.41]) and older adults (2.19 ± 0.29 vs. 0.45 ± 0.18, *p* < 0.001, Hedges’ *g* = −1.33 [−2.07, −0.59]). Finally, significantly enhanced OPA activity was found in older adults compared with young adults during the landmark condition (2.19 ± 0.27 vs. 1.07 ± 0.21, *p* = 0.002, Hedges’ *g* = −1.04 [−1.70, −0.39]) but not the control condition.

## Discussion

In the present exploratory fMRI study, age-related differences in landmark-based navigation were investigated using a simple Y-maze paradigm. The task was designed to limit the influence of mnemonic and motor components to a maximum to gain a preliminary understanding of the neural bases subtending visual-spatial cue reliance in young and healthy older adults.

### Behavior

Well-established findings were corroborated by our results, showing that older adults had lower scores than their younger counterparts on visuospatial cognitive neuropsychological measures, including the perspective-taking, 3D mental rotation, and Corsi block-tapping tasks (Ohta et al., 1981; Clancy Dollinger, 1995; Iachini et al., 2005; Techentin et al., 2014). Moreover, visuospatial memory and perspective-taking ability were associated with measures of navigational behavior. These tests are indeed known to be good predictors of general navigation skills and their decline with age may account in part for older adults’ deficient navigation performance (Zhong and Moffat, 2016). Notably, perspective-taking, mental rotation, and visuospatial memory are important abilities for spatial learning and the dynamic manipulation of sensory information during navigation (Allen et al., 1996; Kozhevnikov et al., 2006; Meneghetti et al., 2018; Muffato et al., 2020).

Consistent with past literature, older subjects’ navigation performances were significantly poorer than young subjects’, with a bias for response-based strategies in older adults (Moffat and Resnick, 2002; Bohbot et al., 2012; Harris and Wolbers, 2012; Rodgers et al., 2012; Gazova et al., 2013; Wiener et al., 2013; Schuck et al., 2015; van der Ham et al., 2015; Zhong and Moffat, 2016; Kimura et al., 2019; Merhav and Wolbers, 2019; Bécu et al., 2020). Indeed, in our study, 52% of younger adults and 83% of older adults preferred a response-based strategy. Our findings are strikingly similar to those from Rodgers et al. (2012) who found that 46% of younger adults and 82% of older adults favored a response-based strategy in a sample of 86 participants. Our results are also in line with those reported by Bohbot et al. (2012) on a large sample of young (*n* = 175) and older (*n* = 125) participants. This navigation strategy preference was associated with older age. However, the possibility that the differential proportion of women in the two age groups (young: 28% vs. old: 59%) partly accounted for the increased use of response-based strategies in older adults cannot be excluded (Perrochon et al., 2018). Notwithstanding these age-related differences, it is important to mention that older participants achieved a high level of performance on the task and made few errors. It can be argued that this result stemmed from the simplicity of the virtual environment that contained a unique junction and three proximal landmarks (Moffat and Resnick, 2002; Caffò et al., 2018). Moreover, both place- and response-based strategies could be used to complete the task.

### Whole-Brain Analyses

Following previous neuroimaging studies looking at the neural bases of spatial navigation, landmark-based navigation recruited an extended network of brain regions (Kuhn and Gallinat, 2014; Spiers and Barry, 2015; Coughlan et al., 2018; Cona and Scarpazza, 2019).

The multiple regression analyses showed that this network spanned posterior structures linked to visuospatial processing. Activation of the left superior occipital gyrus was reported which corresponds to visual area V3A and which is involved in optic flow tracking for visual path integration (Sherrill et al., 2015; Zajac et al., 2019). Also, our landmark-based navigation paradigm elicited activity in the ventral temporal cortex. The latter is known to process high-level visual information such as object quality (Kravitz et al., 2013; Nau et al., 2018). The recruited network also encompassed the posterior section of the hippocampus and the parahippocampal gyrus, brain areas that play a central role in spatial navigation and that are particularly active during immediate retrieval phases of navigation paradigms (Kuhn and Gallinat, 2014; Cona and Scarpazza, 2019). Furthermore, significant activity was found in the angular gyrus, a region of the posterior parietal cortex known to encode landmarks in the environment concerning the self (Ciaramelli et al., 2010; Auger and Maguire, 2018). Our task prompted activation of the prefrontal cortex which is thought to contribute to spatial working memory during active navigation (Wolbers and Hegarty, 2010; Ito, 2018). It thus appears that accurate landmark-based navigation required the integration of objects within a first-person framework and the maintenance of such representations in working memory (Sack, 2009; Seghier, 2012; Miniaci and De Leonibus, 2018). Finally, lobule VI and the vermis of the right cerebellum were found to be activated. This finding is in accordance with the cerebellum’s postulated role in cognitive aspects of spatial navigation (Rochefort et al., 2013). We must nonetheless acknowledge the eventuality that cerebellar activity reflected sensory-motor processing such as the degree of motor learning or eye and finger movements (Bo et al., 2011; Iglói et al., 2015).

Of interest, whole-brain analyses for the contrast [Landmark > Control] revealed that young adults recruited the cortical projections of the central visual field in posterior occipital regions (MNI coordinates left: *x* = −30, *y* = −97, *z* = −7 and right: *x* = 30, *y* = −88, *z* = −10). The latter brain area is dedicated to fine-grained visual perception such as object recognition (Wandell et al., 2005; Kauffmann et al., 2014). Additionally, group comparisons revealed that young subjects had more activity in the anterior section of the inferior temporal gyrus than older subjects. As mentioned previously, the anterior temporal cortex is critical for perceptual recognition and visual object processing (Litman et al., 2009). Our findings resonate with recent evidence highlighting deficient fine-grained processing of sensory information in older adults and emphasize the importance of acute object discrimination for landmark-based navigation and episodic memory (Burke et al., 2018; Greene and Naveh-Benjamin, 2020). Taken together, the above results suggest that brain regions involved in the representation of fine-grained information may be disrupted in older age. Further research is warranted to determine whether the age-related decline in orientation skills could stem from the less efficient processing of visual-spatial cues.

Worthy of note, the differential patterns of neural activity observed in the young and older participant groups may be partially due to age-related cognitive and motor differences. Although the duration of the familiarization phase was tailored to each subject’s needs and we controlled for response device use, the potential influence of older adults’ lesser familiarity with new technologies and declining executive functions cannot be omitted. For example, the lack of activity elicited by the contrast [Landmark > Control] in older subjects could reflect the deficient integration of new instructions when switching between tasks (Hirsch et al., 2016). Additionally, the longer time necessary to reach the visible goal in the control condition along with the greater frontal activations elicited by the contrast [Control > Fixation] in older participants suggests a possible contribution of age-related executive impairment. These results hint at the possibility that the control condition was cognitively more demanding for older participants than for young participants.

A higher level of recruitment of superior parietal regions was detected in older adults compared to young participants from the contrast [Landmark > Fixation]. The aforementioned association between angular gyrus activity and first-person navigation may provide a plausible explanation for the response strategy bias in the older adult group. This is consistent with the observed age-related reduction in temporal activity. Indeed, changes in strategy preference with advancing age have been extensively documented and they are thought to be mediated by a shift from the hippocampal regions towards other cerebral structures such as the parietal cortex (Rodgers et al., 2012; Wiener et al., 2013). Within this framework of interpretation, older adults’ increased cerebellar activity could also reflect a change in strategy preference as recent evidence has implicated the cerebellum in the mediation of response-based strategies (Iglói et al., 2015). Older participants further displayed enhanced activation of frontal cortices. Various authors have stressed the impact of age-related modifications in the prefrontal cortex on hippocampal and striatal dynamics, which could contribute to impaired strategy implementation and switching (Lester et al., 2017; Goodroe et al., 2018; Zhong and Moffat, 2018). In contrast to previous studies that reported striatal activity during response-based navigation, our results did not show increased striatal activity in the older adult group (Konishi et al., 2013; Schuck et al., 2015). Such a difference may be explained by the high proportion of young adults using response-based strategies in our task.

### Scene-Selective Regions Analyses

Given the predominant role of visual perception in human spatial navigation (Ekstrom, 2015; Nau et al., 2018), there has been a heightened interest in the PPA, RSC, and OPA and their respective contributions to landmark processing (Epstein et al., 2017; Julian et al., 2018). It is key to specify that there were no age-related differences in the activity of scene-selective regions when looking at the contrast [Landmark > Control]. However, a seemingly augmented activation of the OPA in older adults along with age-related differences during the control condition led us to conduct further exploratory analyses with the fMRI contrasts [Landmark > Fixation] and [Control > Fixation]. Interestingly, the OPA was more activated than the PPA and RSC in both landmark and control conditions across age groups. This finding fits well with the OPA’s postulated implication in coding navigational affordances and visible paths in the environment (Bonner and Epstein, 2017; Patai and Spiers, 2017). Our results pointed to greater OPA activity in older participants compared with younger participants, which is in line with recent work showing higher functional connectivity around the OPA in older adults (Ramanoël et al., 2019). It is essential to note that the OPA activation in our study cannot be attributed to landmark-based navigation *per se* as it was uncovered by comparing neural activity during the landmark condition and fixation. The OPA is known to be sensitive to self-perceived distance and motion (Persichetti and Dilks, 2016) and the extraction of navigational affordances of the local visual scene from a first-person perspective (Bonner and Epstein, 2017). Critically, these self-centered navigation skills are relatively well preserved in healthy aging (Moffat, 2009). In line with the over-activation of the parietal cortex in older adults, one could conceive that the increased OPA activation in the older adult group reflects a compensatory mechanism to offset the reduced activity in the temporal cortex, thus mitigating age-related place learning deficits. As a side note, considering that the OPA has been causally linked to the processing of environmental boundaries (Julian et al., 2016), our result offers a potential explanation for older adults’ preferential reliance on geometric information in an ecological cue conflict paradigm (Bécu et al., 2020). Such possibilities remain highly speculative and further studies are necessary to test these hypotheses specifically.

Surprisingly, no differences were found in the activity of the RSC across age groups. Previous work has demonstrated an age-related decline in RSC activation during spatial navigation tasks (Meulenbroek et al., 2004; Moffat et al., 2006; Antonova et al., 2009). The RSC is known to mediate several cognitive functions pertaining to spatial navigation (Vann et al., 2009; Mitchell et al., 2018) including translation between reference frames and recollection of visual landmarks (Auger et al., 2015). The discrepancy between our results and those from the literature could be explained by the relative simplicity of our task. In contrast to previous research conducted with young adults, our paradigm strived to restrict mnemonic processing and comprised only three stable, salient and simple landmarks located at a single intersection (Wolbers and Büchel, 2005; Auger et al., 2012; Auger and Maguire, 2018). Another probable explanation lies in the idea that functional and structural changes to the RSC could be more pronounced in pathological aging than in normal aging (Fjell et al., 2014; Dillen et al., 2016).

Finally, weak recruitment of the PPA was observed during landmark-based navigation, with no significant difference across age groups. Previous studies have found the PPA to be involved in the encoding of the navigational relevance of objects for orientation (Janzen and van Turennout, 2004) and in landmark recognition (Epstein and Vass, 2014). As previously noted, our virtual environment comprised a small number of simple and non-ambiguous objects, the lack of activity in the PPA is thus unsurprising. Also, two recent studies de-emphasized PPA’s contribution to active navigation and highlighted its specificity for place recognition (Persichetti and Dilks, 2018, 2019).

Research exploring the neural activity within scene-selective regions in the context of aging is still in its infancy. Future studies are needed to better characterize age-related changes in brain areas implicated in processing both the visual and cognitive properties of spatial cues.

### Limitations and Perspectives

The current study has limitations. First and foremost, there is a possibility that the age-related differences in behavior and neural activity during the control condition may have biased the secondary regression analyses. The latter are of exploratory nature and are to be taken with great caution. Second, although the spatial memory component of the landmark condition was limited to a maximum, the idea that the observed neural activations reflect differences in spatial memory processing or task difficulty between the landmark and control conditions cannot be omitted. Furthermore, the age-related differences observed in the control condition emphasize the plausible impact of more general cognitive deficits such as executive dysfunction on landmark-based navigation. Further work specifically designed to disambiguate the role of age-related differences in visuospatial function and other cognitive dimensions should be conducted. These results also highlight the importance of the task chosen as the control condition in virtual spatial navigation paradigms to be relevant to the population(s) of interest. Critically, our findings put forward the question of whether control tasks are still appropriate in studies comparing complex behavior between young and older adults. Such a topic of research demands closer attention. Third, only the retrieval phase was used for the analyses, as the encoding phase proved to be too heterogeneous across participants. It would be of immediate interest to assess the influence of various visuospatial modulations, such as the visibility of spatial cues, on the quality of spatial encoding. FMRI spatial navigation paradigms only allow for visual input signals, however, “real-world” spatial navigation is reliant upon multiple sources of sensory information. Active walking as part of ecological study designs would provide proprioceptive and self-motion feedback signals as well as an improved field of view to participants. Such studies are necessary to complement the present findings. Previous research has indeed shown that navigation performance in older subjects is tightly coupled to the availability of multiple sources of sensory information (Adamo et al., 2012). Finally, future studies should take into consideration the role of sex and they should include an intermediate age group to gain a finer understanding of the neural dynamics subtending spatial navigation across the lifespan (Grön et al., 2000; van der Ham and Claessen, 2020).

## Conclusion

To conclude, the present study sheds light on the possibility that navigational deficits in old age are linked to functional differences in brain areas involved in visual processing and to impaired representations of landmarks in temporal regions. This work helps towards a better comprehension of the neural dynamics subtending landmark-based navigation and it provides new insights on the impact of age-related spatial processing changes on navigation capabilities. We argue that approaching the study of spatial navigation in healthy and pathological aging from the perspective of visuospatial abilities is a critical next step in the field. Neuroimaging methods coupled with VR paradigms open up promising avenues to investigate age-related changes in navigation ability and to evaluate the benefits of training programs on older adults’ autonomy and mobility.

## Data Availability Statement

The datasets presented in this article are not readily available because the datasets generated for this study are available on reasonable request to the corresponding author. Requests to access the datasets should be directed to stephen.ramanoel@univ-cotedazur.fr.

## Ethics Statement

The studies involving human participants were reviewed and approved by Ethical Committee “CPP Ile de France V” (ID_RCB 2015-A01094-45, CPP N°: 16122). The patients/participants provided their written informed consent to participate in this study.

## Author Contributions

SR, MB, CH, and AA: study design. SR and MD: data acquisition and data processing. SR, MD, MB, and AA: manuscript writing. All authors contributed to the article and approved the submitted version.

## Conflict of Interest

The authors declare that the research was conducted in the absence of any commercial or financial relationships that could be construed as a potential conflict of interest.

## Footnotes

^1^All older participants scored 28 or above on the MMSE except one participant who scored 24. We nonetheless decided to include this subject as the extended neuropsychological evaluation was within normal range and no significant changes were detected when removing the participant from the fMRI analyses.

^2^https://unity.com/
