# Supplementary material for: Differential Brain Activity in Regions Linked to Visuospatial Processing During Landmark-Based Navigation in Young and Healthy Older Adults
Source: Front Hum Neurosci. 2020 Oct 29;14:552111. doi: 10.3389/fnhum.2020.552111 (PMC7668216; doi:10.3389/fnhum.2020.552111)
Supplement: Supplementary file 1 [file Table_1.DOCX]

|  |  | | **H** | **BA** | | **k** | **x** | **y** | **z** | **t** | **ES [95% CI]** | | |
| --- | --- | --- | --- | --- | --- | --- | --- | --- | --- | --- | --- | --- | --- |
| Group Analyses  [Control > Fixation] | |  |  |  | | |  |  | |  |  |  |  |
|  |  | |  |  |  | |  |  |  |  |  | | |
| **[Young]** | Cuneus | | R | 17 | 1154 | | 9 | -88 | 11 | 9.49 | 3.26 [2.59, 3.93] | | |
|  | [Superior Occipital Gyrus] | | L | 18 |  | | -12 | -91 | 14 | 8.79 | 2.54 [1.97, 3.10] | | |
|  | [Calcarine Cortex] | |  | 17 |  | | -9 | -88 | 5 | 8.55 | 3.24 [2.49, 3.98] | | |
|  |  | |  |  |  | |  |  |  |  |  | | |
|  | Superior Frontal Gyrus | | L | - | 131 | | -18 | -4 | 59 | 7.13 | 1.12 [0.81, 1.43] | | |
|  | [Precentral Gyrus] | |  | 6 |  | | -21 | -7 | 74 | 5.83 | 2.10 [1.39, 2.80] | | |
|  |  | |  |  |  | |  |  |  |  |  | | |
|  | Superior Frontal Gyrus | | R | 6 | 96 | | 27 | -4 | 62 | 5.61 | 1.54 [1.00, 2.08] | | |
|  | [Precentral Gyrus]  [Middle Frontal Gyrus] | |  |  |  | | 27  27 | -7  -7 | 74  47 | 4.84  4.62 | 1.76 [1.05, 2.48]  0.78 [0.45, 1.11] | | |
|  |  | |  |  |  | |  |  |  |  |  | | |
|  | Superior Parietal Gyrus | | R | 7 | 43 | | 21  18 | -58  -58 | 68  59 | 5.17  4.86 | 2.29 [1.42, 3.16]  1.78 [1.06, 2.50] | | |
|  |  | |  |  |  | |  |  |  |  |  | | |
|  | Superior Parietal Gyrus | | L | - | 94 | | -24 | -55 | 62 | 4.58 | 1.60 [0.91, 2.28] | | |
|  |  | |  |  |  | |  |  |  |  |  | | |
|  |  | |  |  |  | |  |  |  |  |  | | |
| **[Older]** | Superior Occipital Gyrus  [Superior Occipital Gyrus] | | L | 19 | 177 | | -18  -18 | -88  -91 | 26  14 | 7.23  6.46 | 2.92 [2.13, 3.71]  2.39 [1.67, 3.12] | | |
|  | [Middle Occipital Gyrus] | |  |  |  | | -24 | -85 | 14 | 5.12 | 1.97 [1.21, 2.72] | | |
|  |  | |  |  |  | |  |  |  |  |  | | |
|  | Lingual Gyrus | | L | 18 | 164 | | -12 | -82 | -7 | 6.95 | 2.40 [1.72, 3.07] | | |
|  | [Occipital Fusiform Gyrus] | |  |  |  | | -24 | -76 | -7 | 5.99 | 2.22 [1.49, 2.95] | | |
|  |  | |  |  |  | |  |  |  |  |  | | |
|  | Superior Occipital Gyrus  [Superior Occipital Gyrus] | | R | 39 | 189 | | 30  21 | -79  -85 | 23  20 | 6.23  5.20 | 2.48 [1.70, 3.26]  2.39 [1.67, 3.12] | | |
|  | [Middle Occipital Gyrus] | |  | 19 |  | | 27 | -76 | 32 | 4.99 | 2.06 [1.25, 2.86] | | |
|  |  | |  |  |  | |  |  |  |  |  | | |
|  | Precentral Gyrus | | R | 6 | 130 | | 30 | -7 | 50 | 5.42 | 1.31 [0.84, 1.78] | | |
|  | [Superior Frontal Gyrus] | |  |  |  | | 21 | -1 | 68 | 5.41 | 1.60 [1.02, 2.19] | | |
|  |  | |  |  |  | |  |  |  |  |  | | |
|  | Superior Parietal Gyrus | | L | 5 | 26 | | -27 | -43 | 53 | 5.41 | 1.15 [0.73, 1.57] | | |
|  | [Precuneus] | |  | 7 |  | | -18 | -49 | 56 | 4.79 | 1.01 [0.60, 1.43] | | |
|  |  | |  |  |  | |  |  |  |  |  | | |
|  | Lingual Gyrus | | R | 18 | 27 | | 18 | -85 | -7 | 4.87 | 2.32 [1.38, 3.25] | | |
|  | [Calcarine Cortex] | |  | 17 |  | | 12 | -88 | -1 | 4.35 | 2.49 [1.37, 3.62] | | |
|  |  | |  |  |  | |  |  |  |  |  | | |
|  | Precentral Gyrus | | L | - | 30 | | -33 | -7 | 65 | 4.83 | 2.12 [1.26, 2.98] | | |
|  | [Superior Frontal Gyrus]  [Superior Frontal Gyrus] | |  |  |  | | -24  -24 | -4  -4 | 74  65 | 4.28  4.27 | 2.39 [1.29, 3.48]  1.77 [0.96, 2.59] | | |
|  |  | |  |  |  | |  |  |  |  |  | | |
|  | Superior Parietal Gyrus | | L | 7 | 17 | | -15 | -67 | 50 | 4.75 | 2.00 [1.17, 2.82] | | |
|  | [Precuneus] | |  |  |  | | -12 | -76 | 53 | 4.44 | 3.09 [1.72, 4.45] | | |
|  |  | |  |  |  | |  |  |  |  |  | | |
|  | Superior Frontal Gyrus | | L | 6 | 26 | | -9 | -1 | 71 | 4.68 | 1.47 [0.85, 2.08] | | |
|  | [Supplementary Motor Gyrus] | |  |  |  | | -9 | -10 | 68 | 4.55 | 1.21 [0.69, 1.73] | | |
|  |  | |  |  |  | |  |  |  |  |  | | |
|  | Superior Parietal Gyrus | | R | 40 | 20 | | 36 | -37 | 41 | 4.56 | 1.31 [0.75, 1.87] | | |
|  |  | |  |  |  | |  |  |  |  |  | | |

**Table S1**. Cerebral regions whose activity for the contrast [Control > Fixation] was elicited by within-group analyses (total intracranial volume was included as a covariate). The statistical threshold was defined as p < 0.05 FWE-corrected for multiple comparisons at voxel-level with an extent voxel threshold set at 10 voxels. For each cluster, the region with the maximum t-value is listed first and other regions in the cluster are listed underneath [in square brackets]. Montreal Neurological Institute (MNI) coordinates (x, y, z) of the peak and number of voxels (k) of clusters are also shown. H = hemisphere; R = right hemisphere; L = left hemisphere; BA = Brodmann area; ES = effect size; CI = confidence interval.
