# Supplementary material for: Differential Brain Activity in Regions Linked to Visuospatial Processing During Landmark-Based Navigation in Young and Healthy Older Adults
Source: Front Hum Neurosci. 2020 Oct 29;14:552111. doi: 10.3389/fnhum.2020.552111 (PMC7668216; doi:10.3389/fnhum.2020.552111)
Supplement: Supplementary file 2 [file Table_2.DOCX]

|  |  | | **H** | **BA** | **k** | **x** | **y** | **z** | | **t** | | **ES [95% CI]** | | |
| --- | --- | --- | --- | --- | --- | --- | --- | --- | --- | --- | --- | --- | --- | --- |
| Group Analyses [Landmark > Fixation] | |  |  |  | |  |  | |  | |  | |  |  |
|  |  | |  |  |  |  |  |  | |  | |  | | |
| **[Young]** | Calcarine Cortex  [Lingual Gyrus]  [Calcarine Cortex] | | L  R | 17  18  17 | 741 | -9  -12  12 | -91  -88  -91 | 5  -4  2 | | 8.81  8.28  6.62 | | 3.45 [2.68, 4.22]  2.50 [1.91, 3.10]  2.68 [1.89, 3.48] | | |
|  |  | |  |  |  |  |  |  | |  | |  | | |
|  | Superior Frontal Gyrus | | R | 6 | 128 | 21 | -7 | 68 | | 6.55 | | 1.04 [0.73, 1.35] | | |
|  | [Superior Frontal Gyrus]  [Supplementary Motor Gyrus] | |  |  |  | 24  12 | -7  2 | 56  56 | | 5.71  4.95 | | 0.93 [0.61, 1.25]  0.47 [0.28, 0.65] | | |
|  |  | |  |  |  |  |  |  | |  | |  | | |
|  | Superior Frontal Gyrus | | L | 6 | 79 | -21 | -7 | 65 | | 6.25 | | 1.05 [0.72, 1.38] | | |
|  |  | |  |  |  |  |  |  | |  | |  | | |
|  | Superior Parietal Gyrus | | L | 7 | 14 | -15  -21 | -61  -55 | 71  62 | | 5.24  4.87 | | 1.90 [1.19, 2.61]  1.40 [0.84, 1.97] | | |
|  |  | |  |  |  |  |  |  | |  | |  | | |
|  | Superior Parietal Gyrus | | R | 7 | 68 | 15 | -58 | 59 | | 5.17 | | 1.44 [0.90, 1.99] | | |
|  |  | |  |  |  |  |  |  | |  | |  | | |
|  |  | |  |  |  |  |  |  | |  | |  | | |
| **[Older]** | Superior Occipital Gyrus  [Lingual Gyrus]  [Superior Parietal Gyrus] | | L  R | 18  7 | 4013 | -21  -12  27 | -94  -82  -58 | 17  -7  59 | | 7.90  7.56  7.40 | | 4.46 [3.35, 5.56]  3.04 [2.25, 3.83]  3.40 [2.50, 4.30] | | |
|  | Superior Frontal Gyrus  [Precentral Gyrus]  [Superior Frontal Gyrus] | | R | 6 | 261 | 24  39  21 | -7  -13  -4 | 53  56  74 | | 7.38  6.26  5.40 | | 1.48 [1.08, 1.87]  1.35 [0.93, 1.77]  1.82 [1.16, 2.49] | | |
|  |  | |  |  |  |  |  |  | |  | |  | | |
|  | Precentral Gyrus | | L | - | 855 | -39 | -13 | 56 | | 7.30 | | 1.84 [1.35, 2.33] | | |
|  | [Supplementary Motor Gyrus]  [Precentral Gyrus] | | R  L | 6 |  | 6  -54 | -4  5 | 56  35 | | 6.10  6.00 | | 1.17 [0.80, 1.55]  1.46 [0.98, 1.94] | | |
|  |  | |  |  |  |  |  |  | |  | |  | | |
|  | Cerebellum | | L | - | 40 | -15 | -52 | -46 | | 6.70 | | 0.95 [0.67, 1.23] | | |
|  | Fusiform Gyrus | | R | 36 | 81 | 33 | -37 | -13 | | 5.34 | | 0.68 [0.43, 0.93] | | |
|  | [Cerebellum] | |  |  |  | 21  15 | -46  -49 | -19  -13 | | 4.92  4.79 | | 0.73 [0.44, 1.02]  0.79 [0.47, 1.11] | | |
|  |  | |  |  |  |  |  |  | |  | |  | | |
|  | Precentral Gyrus | | R | 44 | 42 | 54 | 14 | 32 | | 5.11 | | 1.81 [1.12, 2.50] | | |
|  | [Inferior Frontal Gyrus] | |  |  |  | 57 | 8 | 23 | | 5.00 | | 1.34 [0.82, 1.87] | | |
|  |  | |  |  |  |  |  |  | |  | |  | | |
|  | Middle Frontal Gyrus | | L | 9 | 39 | -42  -48  -42 | 38  35  44 | 35  29  23 | | 5.02  4.87  4.28 | | 1.33 [0.81, 1.86]  1.41 [0.84, 1.98]  1.48 [0.80, 2.16] | | |
|  |  | |  |  |  |  |  |  | |  | |  | | |
|  | Cerebellum | | R | - | 35 | 36  33  30 | -43  -43  -49 | -28  -37  -43 | | 5.00  4.60  4.32 | | 1.11 [0.68, 1.55]  1.10 [0.63, 1.57]  0.57 [0.31, 0.83] | | |
|  |  | |  |  |  |  |  |  | |  | |  | | |
|  | Middle Frontal Gyrus | | R | 10 | 52 | 33 | 47 | 20 | | 4.91 | | 1.05 [0.63, 1.47] | | |
|  | [Superior Frontal Gyrus]  [Middle Frontal Gyrus] | |  |  |  | 33  30 | 53  41 | 29  32 | | 4.56  4.41 | | 1.73 [0.98, 2.47]  1.23 [0.68, 1.77] | | |
|  |  | |  |  |  |  |  |  | |  | |  | | |
|  | Cerebellum | | R | - | 18 | 12 | -64 | -40 | | 4.65 | | 0.74 [0.43, 1.05] | | |
|  |  | |  |  |  |  |  |  | |  | |  | | |

**Table S2**. Cerebral regions whose activity for the contrast [Landmark > Fixation] was elicited by within-group analyses (total intracranial volume was included as a covariate). The statistical threshold for cluster was defined as p < 0.05 FWE-corrected for multiple comparisons with an extent voxel threshold set at 10 voxels. For each cluster, the region with the maximum t-value is listed first and other regions in the cluster are listed underneath [in square brackets]. Montreal Neurological Institute (MNI) coordinates (x, y, z) of the peak and number of voxels (k) of clusters are also shown. H = hemisphere; R = right hemisphere; L = left hemisphere; BA = Brodmann area; FWE = family-wise error; ES = effect size; CI = confidence interval.
