# Supplementary figures and images for: Differential Brain Activity in Regions Linked to Visuospatial Processing During Landmark-Based Navigation in Young and Healthy Older Adults
Source: Front Hum Neurosci. 2020 Oct 29;14:552111. doi: 10.3389/fnhum.2020.552111 (PMC7668216; doi:10.3389/fnhum.2020.552111)

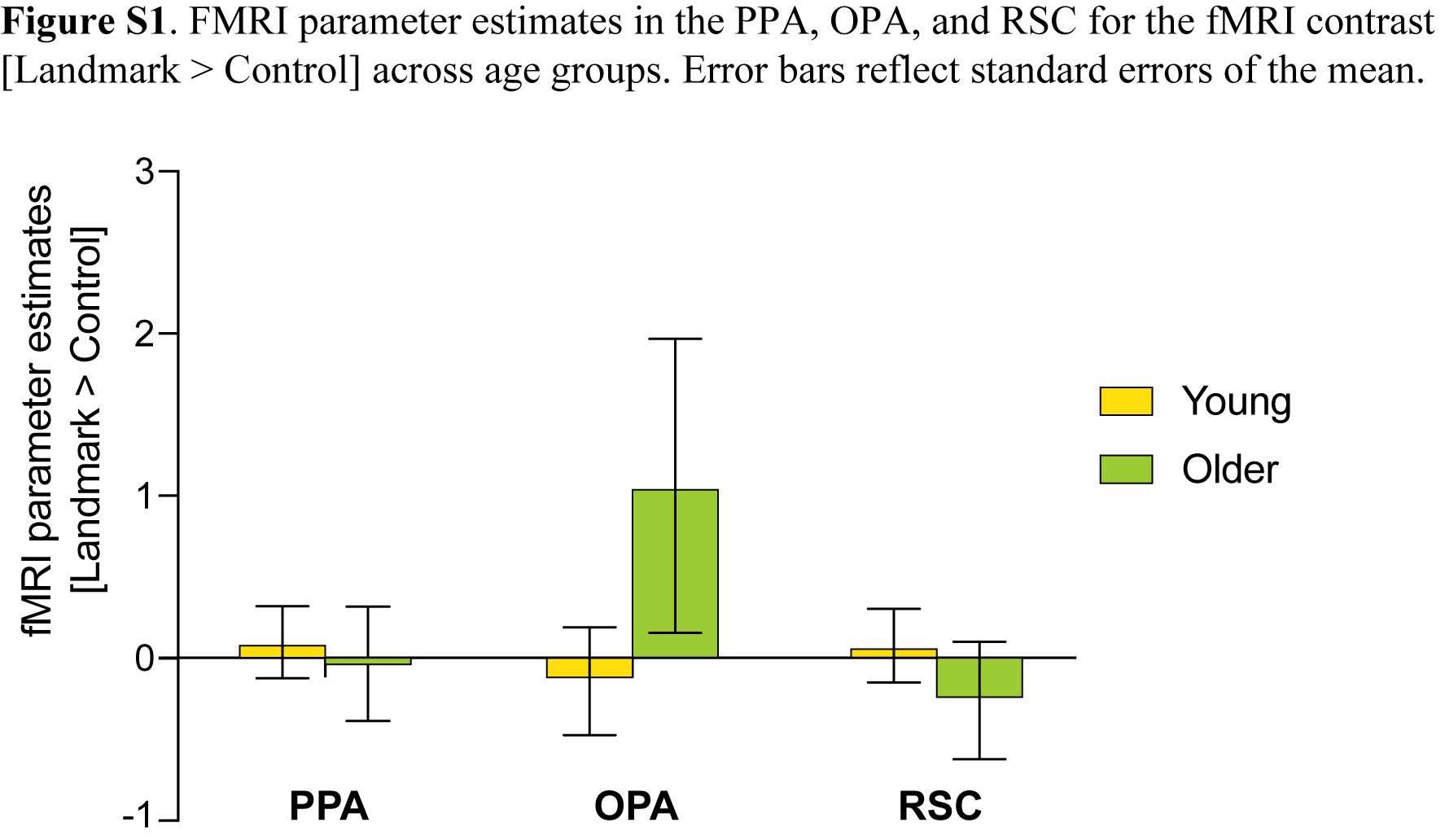

Supplement: Supplementary file 3 [file Image_1.TIF]
